# Somatotype is More Interactive with Strength than Fat Mass and Physical Activity in Peripubertal Children

**DOI:** 10.2478/v10078-011-0063-4

**Published:** 2011-10-04

**Authors:** Carlos Marta, Daniel A. Marinho, Aldo M. Costa, Tiago M. Barbosa, Mário C. Marques

**Affiliations:** 1 Department of Sports Sciences, Polytechnic Institute of Guarda. Education, Communication and Sport School (IPG), Guarda, Portugal; 2 Research Centre for Sports Sciences, Health and Human Development (CIDESD), Vila Real, Portugal; 3 Department of Sport Sciences, University of Beira Interior (UBI), Covilhã, Portugal; 4 Department of Sport Sciences, Exercise and Health, University of Trás-os-Montes and Alto Douro (UTAD), Vila Real, Portugal; 5 Department of Sports Sciences, Polytechnic Institute of Bragança, Bragança, Portugal

**Keywords:** physical fitness, motor performance, strength, anthropometric

## Abstract

The purpose of this study was to analyse the interaction between somatotype, body fat and physical activity in prepubescent children. This was a cross-sectional study design involving 312 children (160 girls, 152 boys) aged between 10 and 11.5 years old (10.8 ± 0.4 years old). Evaluation of body composition was done determining body mass index and body fat by means of skin-fold measurements, using the method described by Slaughter. Somatotype was computed according to the Carter’s method. Physical activity was assessed with the Baecke questionnaire. The physical activity assessment employed sets of curl-ups, push-ups, standing broad jump, medicine ball throw, handgrip strength and Margaria-Kalamen power stair. There were negative associations for body fat, endomorphy and mesomorphy with curl-ups, push-ups and broad jump tests and positive associations with ball throw, handgrip strength and Margaria-Kalamen power tests. The associations for ectomorphy were the inverse of those for endomorphy and mesomorphy. Non obese children presented higher values for curl-ups, push-ups and standing broad jump. In medicine ball throw, handgrip strength and Margaria-Kalamen power test obese children presented higher scores, followed by children who were overweight. The mesoectomorphic boys and ectomesomorphic girls performed higher in all tests. The morphological typology presented more interactions with strength than % of body fat and physical activity. These data seem to suggest that the presence/absence of certain physical characteristics is crucial in the levels of motor provision in prepubescent children.

## Introduction

Children with high levels of motor competence are more active, more capable (Castelli and Vale, 2007) and spend less time on sedentary tasks (Wrotniak et al., 2006). On the other hand, improvement in the motor proficiency of children can also influence levels of habitual physical activity beyond school age, creating expectations of future maintenance of active lifestyles (Sharkey, 2002; Andersen et al., 2004) and is thus indispensable to potential decisions influencing the promotion of health (Stodden et al., 2008). Health-related fitness includes, besides others, aerobic endurance, muscular strength, and flexibility (Hands et al., 2009). On this, most studies on physical fitness have focused specially on aerobic capacity neglecting, among for instance, neuromotor fitness based on muscular strength (Cepero et al., 2011). Some studies reported positive associations between physical activity in children and adolescents with performance on tests of muscular strength and muscular endurance (Lennox et al. 2008; Martínez-Gómez et al., 2011). Added to that, an evolution of muscular strength skills throughout adolescence associated with higher levels of physical activity were also described (Zac and Szopa, 2001). Others, by contrast, report no significant associations between physical activity and performance in similar tests (Hands et al., 2009). It is also possible to find studies that negatively relate body fat with tests of strength and muscular endurance (Castro-Pistro et al. 2009; Dumith et al., 2010) or, conversely, a positive relationship in tests such as ball throwing or handgrip strength (Artero et al., 2010; D’Hondt et al. 2009).

Somatype assessment may be used to describe changes in the human physique over the lifespan or as a result of physical activity and has been found to be inherited to a greater extent than body mass index (Reis et al., 2007). Yet, there are few studies that link somatype with muscular strength in young people, with the exception of the recent studies by Jakšić and Cvetkovic (2009) and Shukla et al. (2009) relating this exclusively to the standing broad jump and curl-ups. Currently, efforts to promote physical fitness levels in the young ought to be a priority (Cepero et al., 2011), but clearly these cannot exceed the limits imposed by genotype, i.e., the manifestation of genetic determinism; just as important as the dimensional values are their relative degrees of presence, observed from the morpho-constitutional perspective (Malina and Bouchard, 1991). We can define the morphological typology as a complex entity that describes the overall configuration of the body, as opposed to individual anatomical characteristics (Malina and Bouchard, 1991). It is therefore pertinent to examine, in addition to the correspondence between physical activity, body composition and performance in tests of muscle strength and endurance, the correlation between somatotype and any such tests.

Therefore, the purpose of the present study was to analyze the relationship between physical activity, body fat, and somatotype with performance in tests of strength and muscular endurance. An additional objective is to find which of the variables is most interactive with the muscular strength and endurance parameters selected. It was hypothesized that there is some kind of relationship between physical activity, body fat and somatotype with muscular strength and muscular endurance performances.

## Material and Methods

### Sample

The sample, cross-sectional in type, consisted of 312 prepubescent children (160 girls, 152 boys) who volunteered for this study. The age, height and weight of the whole sample were: 10.8 ± 0.4 years, 1.45 ± 0.08 m, 40.0 ± 8.7 kg, respectively (girls: 10.8 ± 0.4 years, 1.44 ± 0.07 m, 38.9 ± 8.5 kg; boys: 10.8 ± 0.4 years, 1.45 ± 0.09 m, 41.2 ± 8.8 kg). Descriptive data of the percentage of fat mass (%FAT), body mass index (BMI) endomorphy (ENDO), mesomorphy (MESO), ectomorphy (ECTO), physical activity index (PA) and muscle strength variables are presented in Table 1.

Both boys and girls were in Tanner stages 1–2. Participants’ parents provided their written and informed consent and the procedures were approved by the institutional review board following the Helsinki Declaration.

### Procedures

Parameters of body fat, somatotype, level of physical activity and physical fitness were evaluated for all subjects participating in the study. For anthropometric measurements the participants were barefoot and wore only underwear. Body weight was measured using the standard digital floor scale (Seca 841), body height using a precision stadiometer (Seca 214), and skinfolds using a skinfold caliper. For perimeter measurement a circumference tape was used (Seca 200).

It was assessed the bi-condilofemoral diameter and the leg diameter (Campbell, 20, RossCraft, Canada). In the evaluation of body composition, body mass index (BMI) and body fat (%FAT) were calculated using the skinfold method described by Slaughter et al. (1988). Cohort groups were defined based in the body mass index according to the cut-off values suggested by Cole et al. (2000). The definition of morphological typology (TYPE) used the method described by Heath-Carter (1971), while the evaluation of biological maturation followed the sexual maturation stages of Tanner (1962). Individuals selected were self-evaluated as being in stages 1 and 2. The index of physical activity (PA) was measured using the Baecke et al. (1982) questionnaire. For the assessment of physical fitness, motor tests were chosen to include the assessment of muscle strength and endurance (curl-ups and push-ups: the score was the number of correct curl-ups performed at a cadence of 20 curl-ups per minute, i.e., 1 curl-up every 3 seconds), explosive strength (standing broad jump and medicine ball throw 2 kg: the score was the the furthest distance), isometric strength and anaerobic endurance (hand-grip strength - using a Jamar hydraulic hand dynamometer of 000-200 lbs: three trails were given for each hand separately and the score was recorded in kg) and muscular power (Margaria-Kalamen power stair test: Power = body mass (kg) × vertical distance between steps). The test-retest reliability, as shown by the intraclass correlation coefficient (ICC) was between 0.91 and 0.94 for all measures.

### Statistics

Normality of distribution was checked by applying the Kolmogorov-Smirnov tests (SPSS 17.0). Statistical analysis used the Kruskal-Wallis test in the comparison between groups. Relationships between variables was performed with the Spearman correlation. Interaction between the variables referred to the General Linear Model, MANOVA. The statistical significance was set at p ≤ 0.05.

## Results

There were significant negative associations between % FAT, endomorphy (ENDO) and mesomorphy (MESO) with performance on tests of curl-ups, push-ups and standing broad jump, and positive associations with medicine ball throw, handgrip strength and M-K tests. In the opposite direction, ectomorphy (ECTO) was negatively associated with left handgrip strength, and positively with curl-ups, push-ups and standing broad jump. With the exception of left handgrip strength, for which there were no significant correlations, PA was positively associated with all the variables of muscle strength and endurance (Table 2).

Comparing groups with different BMI, one can observe significant differences between normal weight, overweight, and obese children on curl-ups, push-ups, standing broad jump, medicine ball throw, handgrip strength and M-K power test (Table 3). Normal weight children presented higher performance on curl-ups, push-ups and standing broad jump, followed by children who were overweight. In medicine ball throw, handgrip strength and M-K power test, obese children presented higher scores, followed by children who were overweight (in boys, girls, and whole sample). MANOVA results showed that the variable TYPE presented more interactions with the muscle strength and endurance variables than % FAT and PA variables (Table 5).

The comparison between groups of different TYPE showed significant differences in the curl-ups, push-ups, standing broad jump, handgrip strength and M-K power test (Table 4). In addition, the current experiment presented higher performance values for mesoectomorphic boys and ectomesomorphic girls in all tests.

## Discussion

The purpose of this study was to examine the interaction between physical activity, body fat, somatotype and performance with muscle strength and endurance. The main results suggest that in subjects undergoing pre pubescence, somatotype is a more significantly determining factor than PA and % FAT to the performance in strength tests.

The significant and negative relationship of body fat with body tasks is consistent with results of previous studies that referred to similar relationships in tests of curl-ups (Fogelholm et al. 2008; Castro-Piñero et al. 2009; D’Hondt et al., 2009; Dumith et al., 2010), standing broad jump (Artero et al., 2010; Xianwen et al., 2010), and push-ups (Castro-Piñeroet al., 2009). The current results are also consistent with others researches that report positive associations of body fat with handgrip strength and ball-throwing, as evidenced by studies undertaken by Deforche et al. (2003), Casajús et al. (2007) and Artero et al. (2010), in the hand dynamometry test, and D’Hondt et al. (2009), in the basketball throw. On the other hand, Dumith et al. (2010) found no significant association of body fat with medicine ball throw. In the M-K test, it was not possible to compare the results with other studies in the literature. However, the fact that in the equation for calculating power the numerator must take into account the weight of the subject, which in addition to body fat also includes the muscle mass associated with it, might somehow explain the positive association observed in girls. Concerning the relation of somatotype with physical fitness, it should be stressed-out that, more important than the association of each major component with performance, it is the critical to consider the degree of relative presence of each component, defined by morphological typology.

ENDO was positively related only with handgrip strength, ball-throwing power and the M-K test, these being the same tests in which %FAT had a positive association. These two variables are very close, either in terms of definition, or by the way they are calculated. Here, ENDO expresses the degree of adiposity development (Malina and Bouchard, 1991). So the primary effect of this component in performance will differ depending on the type of task, being a limiting factor in body propulsion and lifting tasks in which body fat plays a similar function. Also Malina and Bouchard (1991) reported that ENDO, unlike the tasks of throwing objects, tends to negatively correlate with performance on most motor tasks, because the absolute lean body mass is more related to these tasks than the relative lean body mass. However, according to the same authors, the correlations between body type and motor performance are generally low and limited in pre pubescence.

MESO reflects muscle development positively associated with strength and motor performance in general (Malina and Bouchard, 1991). This component is only negatively correlated with tests related to the propulsion and lifting of the body, in which tasks ECTO has the advantage, since it is based on weighting index, i.e. the quotient of height by the cube root of body weight (Malina and Bouchard, 1991). While observing a positive influence for MESO in most tests, it is also necessary to consider sexual dimorphism in relation to body type component of the somatotype, reflected in the differences in the values of ECTO and MESO. These differences only begin to be observed and favorable to boys from early adolescence, thus increasing with age, while girls tend to increase the value of ENDO (Malina et al., 2004). If the analysis is carried-out according to the dominant component, the children whom the MESO and ECTO were dominant had the best results in all tests considered.

ECTO reflects linearity and muscular hypotonia (Dumith et al. 2010). On this, there were only positive associations for ECTO with propulsion and lifting body tasks, precisely the reverse of the associations for ENDO and MESO because of the negative effect of body weight in these tasks (Dumith et al. 2010; Xianwen et al., 2010). Regarding left handgrip strength, there is a negative association, since it is a different test, which does not require propulsion or lifting the body.

In the relationship of TYPE with motor performance it appears that meso-ectomorphs and ecto-mesomorphs, i.e. individuals with a predominance of the primary components of ECTO and MESO presented higher performances in all tests considered, which is consistent with the advantage of MESO, noticed in the literature, concerning the tasks that require strength and motor performance in general. By contrast, the advantage of ECTO was found in tasks related to the propulsion and lifting movements of the body. Suchomel (2002) also reported a positive association between the prevalence of these components with the total score of the battery UNIFITT as did Jakšić and Cvetkovic (2009) with the performance analysis in the curl-ups, bent-arm hang and longest jump distance.

The relationship between PA and motor performance corroborates the findings of most studies that report a positive association between PA and muscular strength and endurance as well as overall physical fitness, particularly in the standing broad jump (Lennox et al., 2008; Loko et al. 2003; Wrotniak et al., 2006), medicine ball throw (Loko et al., 2003), push-ups (Lennox et al., 2008, Tovar et al., 2008) and isometric hand-dynamometry (Tovar et al., 2008). Different results were obtained by Hands et al. (2009), who found no significant associations between PA and performance in standing broad jump, curl-ups, hand dynamometry and ball throw. The greatest number of interactions of TYPE with the selected tests highlights the importance of this parameter in muscle strength and endurance in prepubescent children.

In summary, body fat, physical activity and somatotype determine physical fitness levels in children and adolescents. The current study presented similar data, although underlying the main role of the somatotype on muscular strength and resistance in prepubescent children. The data from this study seem to suggest that one cannot exceed the limits imposed by what is a manifestation of genetic determinism, observed from the morpho-constitutional point of view, by which the presence/absence of certain physical traits determines the appropriate levels of motor performance required.

It can be considered as main limitations: (i) there are several other biological and behavior variables that might also determine the muscular strength performance; (ii) it was only applied field tests. Laboratory tests with a higher control standard might present more accurate data.

## Figures and Tables

**Table 1:** Descriptive data of FAT, BMI, ENDO, MESO, ECTO, PA and muscle strength variables

	**Male**		**Female**		**Overall sample**	

Variables	Mean	SD	Mean	SD	Mean	SD
%FAT	22.38	8.65	23.10	6.99	22.75	7.84
BMI	19.46	3.20	18.57	2.93	19.00	3.09
ENDO	3.62	1.71	3.96	1.60	3.79	1.66
MESO	4.69	1.16	3.68	1.09	4.17	1.23
ECTO	2.49	1.45	2.85	1.45	2.67	1.46
PA	8.85	1.09	8.36	0.890	8.60	1.02
Curl-ups	32.96	19.439	25.23	15.01	29.00	17.71
Push-ups	12.42	8.262	8.58	6.78	10.45	7.77
Broad jump	134.87	23.448	119.33	21.98	126.90	23.97
Medicine ball throw	244.80	36.893	219.72	37.79	231.94	39.35
Handgrip strenght R	18.18	3.987	16.903	4.49	17.52	4.29
Handgrip strenght L	16.90	3.801	15.62	4.23	16.24	4.07
M-K power stair test	42.71	13.499	34.667	12.22	38.58	13.45

**Table 2:** Correlations between % of FAT, ENDO, MESO, ECTO, PA and strength variables

		**Male**		**Female**		**Overall sample**	

	**Variables**	**rho**	**p**	**rho**	**p**	**rho**	**p**
**% FAT**	**Curl-ups**	−0.243	0.003^**^	−0.274	0.000^**^	−0.274	0.000^**^
	**Push-ups**	−0.504	0.000^* *^	−0.303	0.000^**^	−0.411	0.000^**^
	**Broad jump**	−0.486	0.000^**^	−0.253	0.001^**^	−0.378	0.000^**^
	**Medicine ball throw**	0.209	0.010^*^	0.193	0.014^*^	0.162	0.004^**^
	**Handgrip strenght R**	0.316	0.000^**^	0.171	0.030^*^	0.232	0.000^**^
	**Handgrip strenght L**	0.338	0.000^**^	0.155	0.050^*^	0.234	0.000^**^
	**M-K power stair test**	0.056	0.490	0.169	0.032^*^	0.073	0.201

**ENDO**	**Curl-ups**	−0.246	0.002^**^	−0.277	0.000^**^	−0.294	0.000^**^
	**Push-ups**	−0.506	0.000^**^	−0.308	0.000^**^	−0.427	0.000^**^
	**Broad jump**	−0.468	0.000^**^	−0.168	0.034^*^	−0.349	0.000^**^
	**Medicine ball throw**	0.200	0.014^*^	0.245	0.002^**^	0.159	0.005^**^
	**Handgrip strenght R**	0.326	0.000^**^	0.249	0.002^**^	0.264	0.000^**^
	**Handgrip strenght L**	0.339	0.000^**^	0.229	0.004^**^	0.257	0.000^**^
	**M-K power stair test**	0.048	0.553	0.213	0.007^**^	0.071	0.214

**MESO**	**Curl-ups**	−0.165	0.042^*^	−0.208	0.008^**^	−0.085	0.134
	**Push-ups**	−0.296	0.000^**^	−0.065	0.415	−0.060	0.291
	**Broad jump**	−0.339	0.000^**^	−0.241	0.002^**^	−0.124	0.028^*^
	**Medicine ball throw**	0.213	0.009^**^	0.009	0.907	0.218	0.000^**^
	**Handgrip strenght R**	0.370	0.000^**^	0.024	0.766	0.219	0.000^**^
	**Handgrip strenght L**	0.385	0.000^**^	−0.011	0.889	0.219	0.000^**^
	**M-K power stair test**	0.179	0.028^*^	−0.030	0.705	0.178	0.002^**^

**ECTO**	**Curl-ups**	0.201	0.013^*^	0.156	0.049 ^*^	0.157	0.005^**^
	**Push-ups**	0.366	0.000^**^	0.160	0.043 ^*^	0.219	0.000^**^
	**Broad jump**	0.489	0.000^**^	0.238	0.002 ^**^	0.303	0.000^**^
	**Medicine ball throw**	−0.045	0.582	−0.082	0.301	−0.094	0.098
	**Handgrip strenght R**	−0.145	0.075	−0.048	0.550	−0.103	0.069
	**Handgrip strenght L**	−0.176	0.030^*^	−0.052	0.518	−0.125	0.027^*^
	**M-K power stair test**	−0.045	0.580	−0.126	0.111	−0.104	0.067

**PA**	**Curl-ups**	0.278	0.001^**^	0.098	0.219	0.225	0.000^**^
	**Push-ups**	0.388	0.000^**^	0.091	0.252	0.270	0.000^**^
	**Broad jump**	0.384	0.000^**^	0.257	0.001^**^	0.366	0.000^**^
	**Medicine ball throw**	0.210	0.010^**^	0.089	0.261	0.212	0.000^**^
	**Handgrip strenght R**	0.166	0.040^*^	−0.005	0.953	0.114	0.044^*^
	**Handgrip strenght L**	0.111	0.172	0.002	0.980	0.096	0.091
	**M-K power stair test**	0.306	0.000^**^	0.090	0.260	0.249	0.000^**^

* p< 0.05;

** p< 0.01

**Table 3:** Comparison between different BMI groups with respect to muscle strength variables

	**Male**		**Female**		**Total**	
	
**Variables**	**K**	**p**	**K**	**p**	**K**	**p**
**Curl-ups**	5.041	0.080	5.442	0.066	8.773	0.012^*^
**Push-ups**	20.216	0.000^**^	10.723	0.005^**^	23.445	0.000^**^
**Broad jump**	26.479	0.000^**^	1.078	0.583	14.192	0.001^**^
**Medicine ball throw**	4.694	0.096	8.892	0.012^*^	14.179	0.001^**^
**Handgrip strenght R**	11.526	0.003^**^	8.067	0.018^*^	21.356	0.000^**^
**Handgrip strenght L**	16.031	0.000^**^	8.739	0.013^*^	26.602	0.000^**^
**M-K power stair test**	0.941	0.625	10.750	0.005^**^	10.808	0.004^**^

* p< 0.05;

** p< 0.01

**Table 4:** Comparison between different TYPE groups with respect to strength variables

	**Male**		**Female**		**Total**	
	
**Variables**	**K**	**p**	**K**	**p**	**K**	**p**
**Curl-ups**	15.206	0.055	20.961	0.034^*^	33.267	0.000^**^
**Push-ups**	43.772	0.000^**^	27.985	0.003^**^	67.080	0.000^**^
**Broad jump**	37.300	0.000^**^	25.003	0.009^**^	44.295	0.000^**^
**Medicine ball throw**	8.731	0.365	13.274	0.276	6.764	0.818
**Handgrip strenght R**	11.282	0.186	20.144	0.043^*^	14.404	0.211
**Handgrip strenght L**	12.442	0.133	27.964	0.003^**^	17.019	0.107
**M-K power stair test**	2.760	0.949	29.445	0.002^**^	20.754	0.036^*^

* p<0.05;

** p< 0.01

**Table 5:** Interaction of morphological type (TYPE), body fat (% FAT) and physical activity (PA) with strength variables: GLM-MANOVA test

		**Male**		**Female**		**Total**	
		
**Variables**		**F**	**p**	**F**	**p**	**F**	**p**
Curl-ups	%FAT			2.674	0.035 ^*^		
	TYPE					2.713	0.002 ^**^
Push-ups							
	%FAT	4.205	0.007 ^**^				
	TYPE					2.909	0.001^**^
Broad jump							
	PA					4.383	0.037^*^
Med ball throw	%FAT					2.601	0.037^*^
H grip strenght R	TYPE			2.135	0.023^*^		
H strenght L	TYPE			2.920	0.002^**^		
	TYPE			3.018	0.001^**^	2.408	0.007^**^
M-K power stair							
	%FAT					2.762	0.028^*^

Legend: H: hangrip; Med: medicine;

* p< 0.05;

** p< 0.01
